# Modulation of Beta-Amyloid-Activated Primary Human Neutrophils by Dietary Phenols from Virgin Olive Oil

**DOI:** 10.3390/nu15040941

**Published:** 2023-02-14

**Authors:** Fernando Rivero-Pino, Elena Grao-Cruces, Soledad Lopez-Enriquez, Gonzalo Alba, Elvira Marquez-Paradas, Carmen M. Claro-Cala, Consuelo Santa-Maria, Sergio Montserrat-de la Paz

**Affiliations:** 1 Department of Medical Biochemistry, Molecular Biology, and Immunology, School of Medicine, Universidad de Sevilla, 41009 Seville, Spain; 2 Department of Pharmacology, Pediatry, and Radiology, School of Medicine, Universidad de Sevilla, 41009 Seville, Spain; 3 Department of Biochemistry and Molecular Biology, School of Pharmacy, University of Seville, 41012 Seville, Spain

**Keywords:** hydroxytyrosol, immunonutrition, neuroinflammation, neutrophils, olive oil, phenols

## Abstract

The defense mechanism against harmful stimuli is inflammation. Indeed, neurodegenerative disorders can arise as a result of a persistent neuroinflammation. Beta-amyloid (Aβ_1-42_) is an early trigger in the origination of Alzheimer’s disease, leading to synaptic and cognitive impairments. Virgin olive oil (VOO) is correlated with a decreased risk of developing immune-inflammatory disorders, but the potential effects of the phenolic fraction (PF) from VOO in the modulation of neuroinflammatory processes in neutrophils remain unknown. In this study, we investigated the ability of the PF to modulate the activation of Aβ_1-42_-stimulated primary human neutrophils, focusing on the expression of gene and surface markers and the release of pro-inflammatory and chemoattractant mediators. Down-regulation of pro-inflammatory cytokine gene expression in Aβ_1-42_-treated neutrophils, among other changes, was reported. Furthermore, pretreatment with PF prevented neutrophil activation. The beneficial effects in the modulation of inflammatory responses show the relevance of VOO to achieve a healthier diet that can help prevent inflammatory diseases.

## 1. Introduction

Virgin olive oil (VOO) is the major source of fatty acids in the traditional Mediterranean diet, and its ingestion is associated with a reduced risk of chronic degenerative diseases, including cardiovascular pathologies, immune-inflammatory disorders, and cancer [1,2,3]. The positive effects of VOO have been historically attributed to the extraordinary oleic acid content, but it is also largely recognized that the minor components like those present in the phenolic fraction (PF) may be of biological relevance [4,5]. In fact, the high concentration of phenolic compounds in the PF from VOO has been reported to be a relevant player in the beneficial effects associated with the Mediterranean diet [6]. Some of these phenolic compounds can exert one or more bioactivities, including anti-inflammatory, antioxidant, antimicrobial, antiproliferative, antiarrhythmic, and vasodilatory effects, as well as the capacity to regulate relevant cellular signaling pathways [7]. The Mediterranean diet, characterized by better cardiovascular health due to VOO, is a food model highly recognized by the scientific community and the World Health Organization.

Inflammation is a physiopathological phenomenon involved in the genesis of numerous diseases as a response of the immune system to injurious stimuli such as tissue injury, infection, or toxicants [8,9]. During physiological inflammation, with a primary role in the clearance of extracellular pathogens, neutrophils and monocytes are recruited from the circulation to sites of inflammation, infiltrated into the affected tissues, and then these cells produce oxidants, complement components, Fc receptors, prostaglandins, cytokines, and chemokines that cooperate to achieve healing and to restore homeostasis [10,11]. Upon identifying cellular injuries, immune cells like microglia, astrocytes, and activated T cells can cause neuroinflammation. Alzheimer’s disease (AD) and other neurodegenerative disorders can arise as a result of a persistent neuroinflammation. In this regard, beta-amyloid (Aβ_1-42_) is an early trigger in the development of AD, leading to synaptic and cognitive deficiency.

As the relationship between VOO and an anti-inflammatory response has been shown [12], it is relevant to unravel the contribution of the PF to these beneficial effects in human neutrophils. This information is key in order to clarify the possible positive impact of phenols during inflammation management and to better understand the complex mechanisms of neuroinflammation and how it is affected by the intake of VOO. The aim of this study was to evaluate whether the PF and its most abundant compound, hydroxytyrosol (HTyr), modulate the activation mediated by Aβ_1-42_ in human neutrophils, in order to unravel the mechanisms behind the beneficial effects of VOO. For this purpose, the Aβ_1-42_-induced expression of pro-inflammatory genes and surface markers and the release of pro-inflammatory cytokines in freshly isolated neutrophils from healthy volunteers exposed to the phenols were evaluated.

## 2. Materials and Methods

### 2.1. Materials

Hydroxytyrosol (HTyr) was purchased from Sigma-Aldrich. The phenolic fraction was extracted from a VOO (picual variety) that was obtained from Ofade Consulting (Seville, Spain). The extraction was carried out as described elsewhere [13] with some modifications [14], freeze dried, and stored at −80 °C until the analyses were carried out. 

### 2.2. Chemical Characterization of PF

Quantitative and qualitative analysis of PF was carried out following the protocol COI/T20/29doc (International Olive Council). The analysis consists of a methanol extraction succeeded by quantification with high-performance liquid chromatography (HPLC) of the supernatant phase filtered. The HPLC system was equipped with a C18 reverse-phase column (4.6 mm × 25 cm), of type Spherisorb ODS-2 (5 mm), 100 Å, with a spectrophotometric UV detector at 280 nm and integrator. The amount of phenols was determined by measuring the areas of the related chromatographic peaks and expressed as mg of compound/kg of oil. 

### 2.3. Blood Collection and Neutrophil Isolation

The rules of good clinical practice were followed when conducting this investigation. The research followed the guidelines set forth in the World Medical Association’s Helsinki Declaration. Buffy coats provided from the Regional Center for Blood Transfusions and the Tejidos Bank of Seville and Huelva were used to harvest neutrophils (S2200035). Human peripheral blood mononuclear cells were isolated by centrifugation on a gradient with Ficoll (Sigma, Madrid, Spain). Neutrophils were isolated by dextran sedimentation in a Ficoll Histopaque gradient (Sigma-Aldrich, St. Louis, MO, USA) and erythrocytes were removed with hypotonic lysis. RPMI 1640 medium supplemented with L-glutamine, penicillin, streptomycin, and 1% heat-inactivated fetal bovine serum was used to suspend the cells after isolation. Trypan blue exclusion was used to ensure that >97% of the neutrophil preparation was viable.

### 2.4. Cytotoxicity Assay

Neutrophils seeded in 96-well plates (1 × 105 cells/well) were incubated for 6 hours with or without different PF or HTyr concentrations (25 and 50 µg/mL). Then, the effect on cell viability was analyzed with 3-(4,5-dimethylthiazol-2-yl)-2,5-diphenyltetrazolium bromide (MTT) colorimetric assay. Cell survival was measured as the percentage of absorbance compared with untreated control cells.

### 2.5. Cell Activation and Exposure to PF

Neutrophils were seeded at a density of 3 × 10^6^ cells/mL and exposed to 500 nM of β-amyloid (Aβ_1-42_, Sigma-Aldrich) in the presence or absence of PF (25 and 50 µg/mL) or HTyr (41 µM) for 6 h. 

### 2.6. Flow Cytometry

Membrane expression of CD16b (PE anti-human CD16b, BD Biosciences, San Jose, CA, USA), CD62L (FITC anti-human CD62L, Miltenyi Biotec, Bisley, UK), CD63 (PE-Cy7 anti-human CD63, BD Biosciences), and CXCR1 (APC anti-human CD181, BD Biosciences) on neutrophils was assessed with flow cytometry (FACS). According to the manufacturer’s instructions, neutrophils (10^6^ cells) were treated with Aβ_1-42_ for 6 h and then incubated with the corresponding antibodies at room temperature, in the dark, for 15 min. Afterwards, the cells were fixed, and the erythrocytes were lysed with 20× volume of FACS lysing solution (BD Bioscience). At the Research, Technology, and Innovation Center (University of Seville), fluorescence intensity in the resulting suspension was measured with a FACS Canto II cytometer (BD Bioscience) with the support of FACS Canto II cell analyzer software (BD Bioscience). Each sample’s mean fluorescence intensity (MFI) was calculated using 10^4^ counted cells. Forward scatterhigh (FSChigh), side scatterhigh (SSChigh), and CD16 high cells were used to gate neutrophils. Expression levels were shown as MFI after being adjusted for nonspecific binding of isotype control antibodies on donor neutrophils.

### 2.7. RNA Isolation and qRT-PCR Analysis

Total RNA was also obtained from cells using Trisure Reagent (Bioline GmbH, Berlin, Germany). RNA quality was evaluated with an A260/A280 ratio in a NanoDrop ND-1000 Spectrophotometer (Thermo Fisher Scientific, Wilmington, DE). RNA (1 µg) was subjected to reverse transcription (iScript, BioRad, Madrid, Spain) following the manufacturer’s indications. Ten ng of the resulting cDNA was employed as a template for real-time polymerase chain reaction (PCR) amplifications. The mRNA levels for individual genes were quantified using real-time PCR in a CFX96 system (BioRad). For each PCR reaction, cDNA template was added to Brilliant SYBR green QPCR Supermix (BioRad, CA, USA) containing the primer pairs for either gene or for glyceraldehyde 3-phosphate dehydrogenase (GAPDH) and hypoxanthine phosphoribosyltransferase (HPRT) as housekeeping genes (Table 1). The average threshold cycle (Ct) values of all the triplicate amplification reactions were used to determine the relative mRNA expression of the candidate genes. Using the conventional 2-(ΔΔCt) technique, the magnitude of change in mRNA expression for candidate genes was determined. All data were reported as a percentage of controls after being adjusted to the content of the endogenous reference genes (GAPDH and HPRT).

### 2.8. Quantification of Cytokine Levels

The cytokine levels of IL-1β, IL-6, IL-8, TNF-α, and IFN-γ in cell supernatants were quantified with enzyme-linked immunosorbent assay (ELISA), according to the indications of the provider (Diaclone, Besançon, France).

### 2.9. Statistical Analysis

Data were assessed using Graph Pad Prism Version 6.1 software (San Diego, CA, USA). The arithmetic mean and standard error of the mean are used to express all values in the figures and text. There were three replicates of each experiment. One-way analysis of variance (ANOVA) was used to determine the statistical significance of any differences in each parameter between the groups, with the Dunnett test serving as the post hoc test. Statistics were considered significant at *p* values less than 0.05.

## 3. Results and Discussion

### 3.1. Characterization of the Phenolic Fraction

The composition of PF is detailed in Table 2. The main components found in the phenol fraction were the aldehyde form of oleuropein aglycone and the dialdehyde form of ligstroside aglycone, followed by HTyr and tyrosol, representing around 67% of the total PF. These results are in line with previous reports aiming to characterize the phenolic fraction of VOO [15]. Phenolic compounds are accumulated during fruit ripening, and both agronomic (i.e., cultivars or environmental factors) and processing factors (e.g., malaxation time) have an impact on their biosynthesis and biotransformation factors [15,16]. For instance, the content of HTyr has been reported to range from 0.28 to 7.57 mg/kg in olive oil in an evaluation of 80 different cultivars [17]. Thus, the content of HTyr in the test item is relatively high. The antioxidant and anti-inflammatory properties of olive phenols were recently reviewed, highlighting the relevance of HTyr [18]. 

In fact, in terms of health properties, the European Food Safety Authority (EFSA) has declared that olive oil phenols contribute to the protection of blood lipids from oxidative stress, referring to 5 mg of HTyr and its derivatives per 20 g of olive oil. The oil evaluated contained 7 mg/20 g; thus, antioxidant activities at least are expected to occur. However, there is not a health claim concerning anti-inflammatory or other properties of VOO’s phenolic compounds because the research until now is still limited.

### 3.2. Effect of PF and HTyr on Neutrophil Viability

Cell survival was determined with MTT assay, and the viability at all doses assessed was >95% (data not shown). It was observed that PF up to 50 µg/mL and HTyr at 41 µM (equivalent concentration of HTyr found in PF at 50 µg/mL) for 6 h had no adverse effects on neutrophil viability. Considering these results, the rest of the assays were carried out at concentrations of 25 and 50 μg/mL. 

### 3.3. Regulation of Myeloperoxidase, Neutrophil Elastase, and Cyclooxygenase-2 Gene Expression

**Figure 1:**
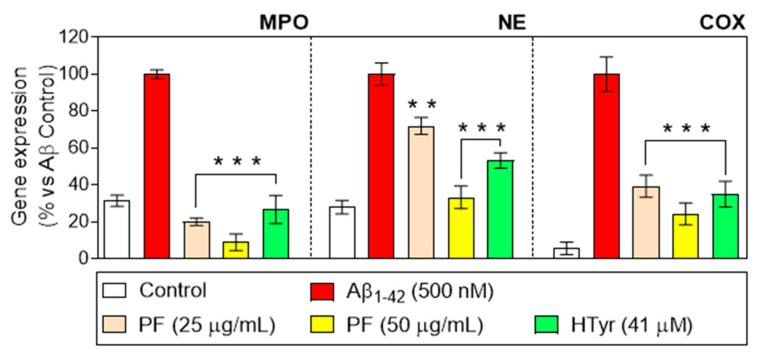
Relative gene expression of myeloperoxidase (MPO), neutrophil elastase (NE), and cyclooxygenase-2 (COX-2) in primary human neutrophils stimulated with Aβ_1-42_ (500 nM), after treatment with the PF from VOO (25 and 50 μg/mL) or hydroxytyrosol (HTyr, 41 μM) for 6 h. Values are means of the three independent experiments in triplicate, with their standard errors represented by vertical bars (** *p* < 0.01; *** *p* < 0.001 vs. Aβ_1-42_-treated cells).

Gene expression following exposure to the PF and pure HTyr was evaluated in the Aβ_1-42_-activated neutrophils. Aβ_1-42_-treated neutrophils up-regulated myeloperoxidase (MPO), neutrophil elastase (NE), and cyclooxygenase-2 (COX-2) gene expression compared with untreated neutrophils (Figure 1). PF at both concentrations of 25 and 50 µg/mL, and HTyr at 41 µM, interfered with the enhanced transcriptional activity of MPO, NE, and COX-2 genes in Aβ_1-42_-treated neutrophils. In the case of PF, the response was observed in a dose-dependent manner. MPO is a local modulator of tissue injury and the subsequent inflammation related to many diseases, which is mostly expressed in neutrophils [19]. In the case of MPO gene expression, the PF led to a decrease lower than the basal levels (control), whereas the HTyr decreased to a lesser extent, indicating that the effects are due to the contribution of different compounds, rather than HTyr being the main contributor.

NE, which can be passively liberated or actively produced, has been correlated with the development of several inflammatory diseases [20], whereas cyclooxygenase-2 (COX-2) expression by the neutrophils results in PGE2 synthesis, which may account for alterations in tissue homeostasis. The regulation of NE seems to be more affected by other phenols present in the fraction rather than HTyr, compared with the other markers evaluated, since at 25 µg/mL the decrease, though significant, is not very relevant. The highest decrease is observed with the PF at 50 µg/mL where the levels are comparable to the control; thus, the increased concentration of phenols might be contributing to this effect. The pattern of gene expression found for COX-2 is similar to the other markers but the difference among samples is not very pronounced, suggesting in this case that HTyr is highly contributing to the regulation of COX-2.

Among the phenolic compounds present in the composition of olive oil, for instance, oleocanthal has shown in vitro inhibition of cyclooxygenase in ranges from 7–100 μM from the prostaglandin-biosynthesis pathway, indicating an anti-inflammatory potential similar to ibuprofen [21]. This study shows that the Aβ_1-42_-mediated increase in MPO expression was effectively prevented by PF and HTyr treatment. According to prior research, the heme enzyme MPO, whose expression is restricted to neutrophils and monocytes, is a key factor in determining how inflammation develops, catalyzing the formation of reactive oxygen intermediates [19]. In addition, MPO is accepted to be a local modulator of tissue injury and the resulting inflammation in many inflammatory diseases. Moreover, in neutrophils COX-2, which leads to pro-oxidant production and tissue damage, contributes to the neutrophils’ self-amplifying recruitment, further amplifying the inflammatory response [22]. In this study, the Aβ_1-42_-induced increase in the COX-2 gene and protein expression was markedly attenuated by the PF or HTyr treatment, suggesting that the phenolic components from VOO might reduce oxidative damage brought on by an inflammatory stimulation in human neutrophils.

Together with MPO, neutrophil azurophil granules contain high amounts of NE, which has been correlated with the development of several inflammatory diseases [20,23,24]. Under normal physiological conditions, this enzyme is controlled by endogenous serine proteinase, but in the presence of pro-oxidant species such as MPO-generated hypochlorous acid, this feedback mechanism is lost. This study suggests that PF and HTyr also have the capacity to inhibit the gene expression of NE in Aβ_1-42_-activated human neutrophils, contributing to the response modulation. These results are in line with Czerwińska et al. [25], who reported that oleacein (an isolated compound from the PF of VOO) was able to inhibit NE release in N-formyl-methionyl-leucyl-phenylalanine–stimulated human neutrophils. In addition, animal studies have shown the anti-inflammatory effects of phenols from olive oil, such as differences in the expression of COX-2, mPGES-1, and iNOS protein, and decreases in the levels of circulatory matrix metalloproteinase (MMP)-3 and pro-inflammatory cytokines in collagen-induced arthritis murine models [26]. The results obtained within this research provide a more in-depth insight into the mechanisms behind the immune response and how it is modulated by the VOO’s polyphenols from a commercial sample and specifically for HTyr.

### 3.4. Down-Regulation of Toll-like Receptor 4 and Pro-Inflammatory Cytokine Gene Expression and Secretion 

**Figure 2:**
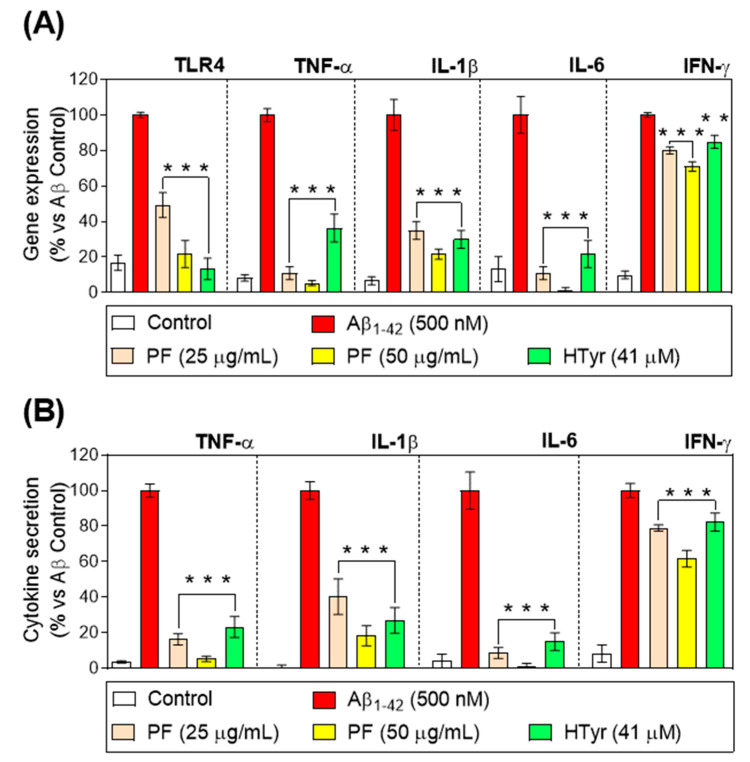
(**A**) Relative gene expression of TLR-4, tumor necrosis factor (TNF)-α, interleukin (IL)-1β, IL-6, and interferon (IFN)-γ. (**B**) Cytokine expression of TNF-α, IL-1β, IL-6, and IFN-γ after the treatment of Aβ_1-42_-stimulated primary human neutrophils with the PF from VOO (25 and 50 μg/mL) or hydroxytyrosol (HTyr, 41 μM) for 6 h. Values are means of the three independent experiments in triplicate, with their standard errors represented by vertical bars (** *p* < 0.01; *** *p* < 0.001 vs. Aβ_1-42_-treated cells).

The stimulation of toll-like receptor (TLR) 4 by Aβ_1-42_ causes the release of vital immunoregulatory and pro-inflammatory cytokines, both of which are necessary to effectively activate the innate immune response. [27]. In this study, an up-regulation of the TLR4 gene expression in Aβ_1-42_-treated human neutrophils was reported, but this was counteracted by the treatment with PF and HTyr for 6 h (*p* < 0.001 vs. Aβ_1-42_-control, Figure 2A). Furthermore, the PF and HTyr significantly decreased the gene expression and production of the pro-inflammatory cytokines TNF-α, IL-1β, IL-6, and IFN-γ at all doses after 6 h (*p* < 0.001 vs. Aβ_1-42_-control, Figure 2B). The regulation of TLR4 is suggested to be due to the HTyr, as it was observed that the decrease was more pronounced when the exposure was performed with the isolated compound. This behavior was different for the rest of the markers in Figure 2. For instance, the gene expression of TFN-α and IL-6 is significantly different from the Aβ_1-42_-treated neutrophils and lower than the decrease effect caused by HTyr. This suggests that the other phenols (Table 2) in the test item are responsible for this counteracting effect. For the other parameters evaluated (IL-1β and IFN-γ) the pattern is similar, where the contribution seems to be balanced among the different phenols, since the HTyr is exerting the same effect as the PF.

**Figure 3:**
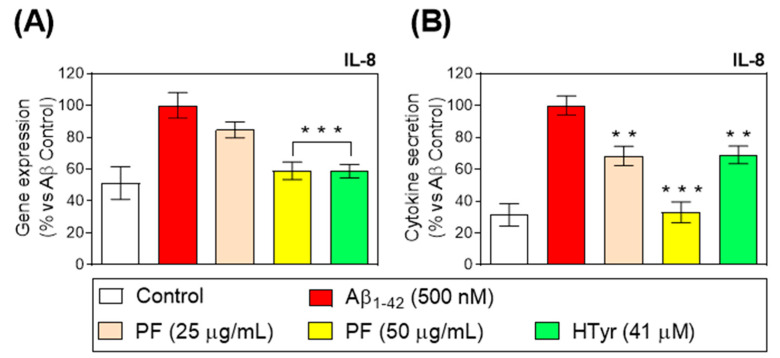
(**A**) Relative gene expression and (**B**) release of IL-8 in Aβ_1-42_-stimulated primary human neutrophils after treatment with the PF from VOO (25 and 50 μg/mL) or hydroxytyrosol (HTyr, 41 μM) for 6 h. Values are means of the three independent experiments in triplicate, with their standard errors represented by vertical bars (** *p* < 0.01; *** *p* < 0.001 vs. Aβ_1-42_-treated cells).

IL-8 was described to be one of the first chemokines triggering neutrophils after secretion by stimulated monocytes [28]. Aβ_1-42_-treated neutrophils up-regulated IL-8 gene expression and secretion compared with untreated neutrophils (Figure 3). In this study, the treatment of Aβ_1-42_-stimulated human neutrophils with the PF and HTyr for 6 h at concentrations up to 50 µg/mL (PF) and at 41 µM (HTyr) resulted in a decrease in the IL-8 gene expression and production by neutrophils. Furthermore, the PF at the highest concentration inhibited IL-8 release in a more significant manner than HTyr at 41 μM (*p* < 0.01; *p* < 0.001 vs. Aβ_1-42_-control, Figure 3).

A great body of evidence has confirmed that human neutrophils are a target but also a source of several pro-inflammatory cytokines [29]. Interestingly, IL-8 is not only the most abundantly secreted cytokine by neutrophils, but also, neutrophils are its primary cellular target [28]. This cytokine activates neutrophils to induce chemotaxis, trigger burst, and degranulation and stimulates neutrophil adhesion to endothelial cells. In the present study, it was found that Aβ_1-42_-mediated IL-8 gene expression/secretion was markedly attenuated by the treatment with PF and HTyr in human neutrophils. This ability of modulating IL-8 secretion was also previously reported by Czerwińska et al. [25], who found that oleacein, the above-mentioned olive oil polyphenol, inhibited IL-8 secretion in stimulated human neutrophils. Moreover, PF and HTyr abrogated the gene expression of TLR in addition to decreasing the gene expression/secretion of key pro-inflammatory cytokines, including TNF-α, IL-1β, IL-6, and IFN-γ in Aβ_1-42_-activated human neutrophils and monocytes. In this regard, one of the relevant events during inflammation is leukocyte infiltration, which is regulated by several chemokines for neutrophils and monocytes, whose release is regulated by iNOS-derived NO [30]. In accordance with the aforementioned cytokines data, the treatment with PF (50 µg/mL) or HTyr (41µM) was able to prevent the induced protein expression of iNOS during the Aβ_1-42_ challenge. 

It was recently reported that HTyr (up to 40 mg/kg) exerted antioxidative and anti-inflammatory activities in the splenic tissue after lipopolysaccharide (LPS)-mediated septic response in mice. In this study, animals ingested HTyr for 10 days, and results showed an enhancement of the survival rate and a decrease in the lactate dehydrogenase level in LPS-challenged mice. In addition, the oxidative damage was reduced as well the production levels of TNF-α, IL-1β, and IL-6. In this study, the mRNA expression of iNOS and NO production also increased [31]. Overall, the results reported are in accordance with our results, which explained the mechanisms behind the neutrophils’ role. In addition, a transcriptomic profile analysis reported the effects of HTyr on the whole-genome expression of endothelial cells under resting or pro-inflammatory conditions, resulting in 599 affected genes in IL-1β–stimulated conditions, highlighting the immunological, inflammatory, proliferative, and metabolic pathways affected [32]. These results help to explain how the body system, depending on the health of the subject, is affected at different levels following the ingestion of bioactive compounds.

### 3.5. Regulation of Metalloproteinases and Peroxisome Proliferator-Activated Receptor-γ Gene Expression

**Figure 4:**
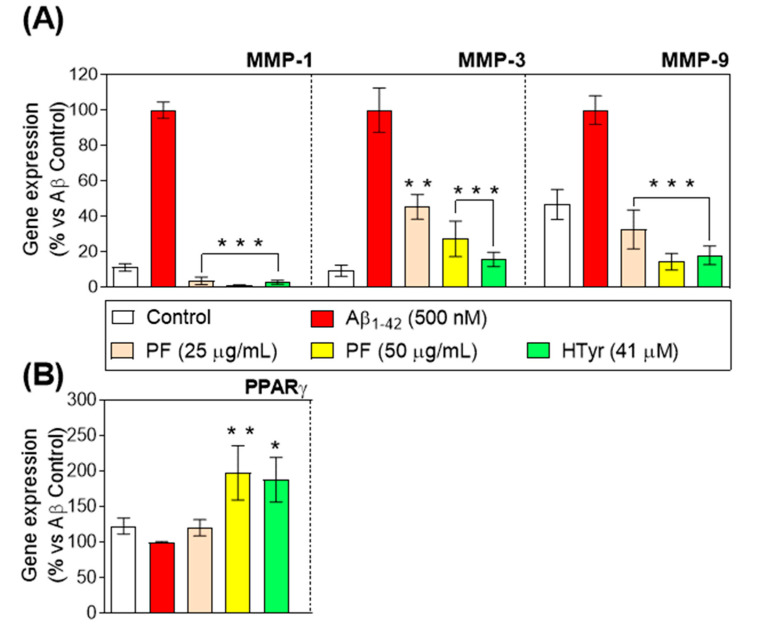
Relative gene expression of (**A**) MMP-1, MMP-3, and MMP-9 and (**B**) PPAR-γ in Aβ_1-42_-stimulated primary human neutrophils treated with the PF from VOO (25 and 50 μg/mL) or hydroxytyrosol (HTyr, 41 μM) for 6 h. Values are means of the three independent experiments in triplicate, with their standard errors represented by vertical bars (* *p* < 0.05; ** *p* < 0.01; *** *p* < 0.001 vs. Aβ_1-42_-treated cells).

Metalloproteinases (MMPs) modulate the events of immune cell development, which are considered relevant for persistent inflammatory response [33]. Therefore, we investigated the influence of PF and HTyr on the gene expression of three major MMPs (MMP-1, MMP-3, and MMP-9). As peroxisome proliferator–activated receptor (PPAR)-γ agonists have been reported to inhibit MMPs and pro-inflammatory cytokines [34], whether the PF and HTyr can affect PPAR-γ gene expression in Aβ_1-42_-treated human neutrophils was also investigated. Aβ_1-42_-treated neutrophils up-regulated MMP gene expression compared with untreated neutrophils. As shown in Figure 4A, the incubation with PF for 6 h produced a significant down-regulation of all MMPs gene expression in Aβ_1-42_-treated human neutrophils. As can be observed, the effects of olive oil phenols on the expression of MMP could be considered highly biologically relevant, as it counteracts the effect, leading to even lower values of expression compared with the control for MMP-1, whereas the effect on MMP-3 and MMP-9 is a partial decrease. The effect of HTyr is very relevant for MMP-3, whereas in the case of MMP-9, results suggest that the effects are due to the different phenols contained as a whole, rather than because of HTyr exclusively. These findings were further endorsed by a significant up-regulation of PPAR-γ gene expression in Aβ_1-42_-treated human neutrophils following the incubation with 50 µg/mL of PF and 41 µM of HTyr (*p* < 0.05; *p* < 0.001 vs. Aβ_1-42_-control, respectively, Figure 4B).

MMPs, especially LPS-induced interstitial collagenase-1 (MMP-1), stromelysin-1 (MMP-3), and gelatinase B (MMP-9), have been described as involved in inflammation via destroying extracellular matrix elements and controlling cytokine signaling by interacting with COX pathways [35,36,37]. These results demonstrate that the PF and HTyr repressed the gene expression of MMP-1, MMP-3, and MMP-9 in human neutrophils, in agreement with other in vitro studies where HTyr, at nutritional concentrations, reduces MMP-9 and COX-2 induction in phorbol-12-myristate-13-acetate (PMA)-activated human monocytes [38]. It was also demonstrated that the PF and HTyr induced PPARγ up-regulation, which, by inhibiting MMP expression in inflamed tissues, plays a crucial part in the dynamic balance between overall matrix synthesis, deposition, and deterioration and affects the expression of pro-inflammatory cytokines [39].

Aβ_1-42_ is one of the key factors for the development of AD, as it can trigger neuronal inflammation. These results suggest that the modified expression of pro-inflammatory genes and surface markers, as well as the release of pro-inflammatory cytokines due to the phenols from VOO, are exerting a positive effect on the modulation of the physiological process leading to neuronal inflammation, and can be helpful in the management of these diseases.

### 3.6. Olive Oil Phenols Prevent Activation of Human Neutrophils

**Figure 5:**
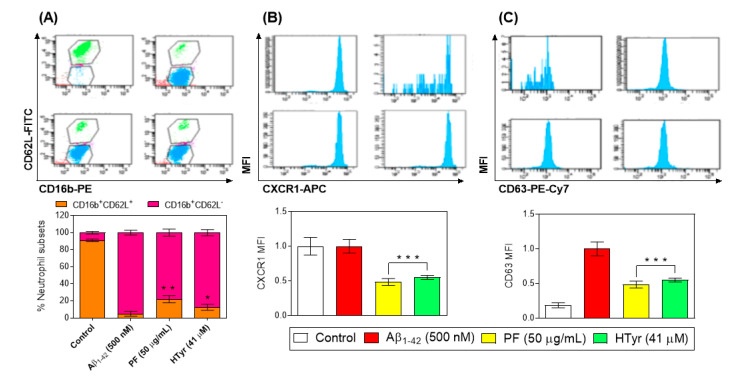
Membrane expression of (**A**) CD16b, (**B**) CXCR1, and (**C**) CD63 in Aβ_1-42_-stimulated primary human neutrophils treated with the PF from VOO (25 and 50 μg/mL) or hydroxytyrosol (HTyr, 41 μM) for 6 h. Values are means of the three independent experiments in triplicate, with their standard errors represented by vertical bars (* *p* < 0.05; ** *p* < 0.01; *** *p* < 0.001 vs. Aβ_1-42_-treated cells).

The neutrophil population in peripheral blood can be discriminated with flow cytometry based on their forward- and side-scatter characteristics. After testing several combinations of antibodies, the best results were obtained by gating the CD16b and CD62L population. When cells were gated, we analyzed them according to their CD16b+ and CD62L- expression. The CD16b + CD62L- population was subjected to other activation markers analysis such as CXCR1 and CD63 (Figure 5). As expected, we found a higher content of activated neutrophils in Aβ_1-42_ -treated cells (*p* < 0.001 vs. Aβ_1-42_-control, Figure 5. Interestingly, we observed a significant decrease after the treatment with HTyr 41 μM compared with PF (50 μg/mL), which does not differ from the control, supporting the evidence that HTyr is one of the main contributors of the bioactivity described for phenols of olive oil.

The innate immune system’s first line of defense is neutrophils, the most prevalent type of leukocyte in human blood [40]. Once activated, they are complex cells able to perform a substantial range of specific functions, and as effector cells of the innate immune response they are capable of regulating many pathological processes, including autoimmunity, cancer, and chronic inflammation. During inflammatory processes, neutrophils can interplay directly or through cytokines and chemokines with other immune cells to modify both the innate and adaptive immune responses [29]. The inflammatory response relies greatly on neutrophil activation, which entails a variety of different processes that lead to the synthesis of cytokines as well as neutrophil migration into tissues [29,41]. CD16b (FcγRIIIb) is exclusively expressed by human neutrophils and undergoes efficient ectodomain shedding upon neutrophil activation and apoptosis [42]. Moreover, neutrophils’ migration from the circulation into an area of inflammation involves L-selectin (CD62L) that is important in the initial attachment of leukocytes to the endothelium [43]. CXCR1 is a closely related receptor that recognizes CXC chemokines including CXCL8 (or IL-8) and CXCL6, and its down-regulation increases neutrophil adhesion and impairs its migration [44]. NE and other luminal proteins are stored in MPO-positive secretory lysosomes/primary granules of neutrophils, which contain an integral membrane protein, CD63, with an adaptor protein-3-dependent granule delivery system [45]. Once the activated population, based on CD16b++CD62L-CXCR1midCD63++ was established, it was found that the presence of PF and HTyr resulted in decreased frequencies of activated neutrophils when compared with Aβ_1-42_-activated neutrophils. Together, these results suggest that the anti-inflammatory properties of the PF and HTyr are not restricted to their antioxidant power, but also involve other properties, including the attenuation of neutrophil activation. 

Beyond in vitro and animal studies, there are also some human studies reporting the potential anti-inflammatory effects of polyphenol-enriched olive oils. Patti et al. [46] reported differences in several parameters, including pro- and anti-inflammatory cytokines in patients with metabolic syndrome and hepatic steatosis, after the intake of 32 g of the test item during 60 days. The intake of 40 mL/day for 9 weeks of oils with different contents of minor polar compounds (706.36 and 485.01 mg/L) was also assessed in patients. Authors indicated an improvement in the biomarkers related to renal function as well as in inflammatory parameters and body composition, among other parameters, supporting the evidence that the phenolic compounds are contributing to the health benefits of this food [47]. However, de Santis et al. [48] recently stated that there is still a gap to solve concerning the correlation between the chemical characterization of VOO compounds and biological activity in humans, which is indispensable to clearly state health claims. The complex composition of olives, specifically regarding their polyphenol content and how it can vary depending on several factors, makes it hard to draw conclusions on the effects of olive oil phenols as such, since the synergy among the different components leads to different physiological responses.

## 4. Conclusions

VOO is known to be a significant bioactive food with a variety of advantageous qualities, and it may be useful in the treatment of various immune-inflammatory illnesses. On top of the fatty acid composition, where oleic acid as a major compound is considered an important contributor to these effects, VOO has other biological minor components that also account for its beneficial activities. The effect of the phenols from VOO on neutrophils, a key cell type in the immune response, was investigated in order to unravel the mechanisms behind the response. Results showed similar effects mediated by both total PF and isolated HTyr in Aβ_1-42_-activated human neutrophils, suggesting that HTyr plays a predominant role in the antioxidant and anti-inflammatory effects of the PF and strengthening the current knowledge concerning the main biological properties attributed to HTyr. Nevertheless, other bioactive substances found in the PF (e.g., tyrosol, pinoresinol, or oleocanthal, among others) may also contribute to PF’s beneficial effects. In fact, earlier studies have referred to these small polyphenols in olive oil as a type of natural substance with anti-inflammatory and antioxidant capabilities. Considering that Aβ_1-42_ is an early trigger in the pathogenesis of AD, leading to synaptic and cognitive impairments, these results show the beneficial effect of phenols in managing the inflammation associated with neuronal diseases, slowing down the development of the process.

Further studies are required to clarify the mechanisms underlying the beneficial effects of PF in the inflammatory process in order to design convenient therapeutic strategies and indications. Nevertheless, these results suggested that olive oil polyphenols have a significant potential to modulate inflammatory conditions distinguished by an over-activation of neutrophils, and thereby they could be an interesting alternative for the management of the inflammatory response.

## Figures and Tables

**Table 1:** Sequences of RT-PCR primers for gene expression analysis.

Target	GenBank Accession Number	Direction	Sequence (5′ → 3′)
TLR4	NM_138554.4	Forward Reverse	TGAGCAGTCGTGCTGGTATC CAGGGCTTTTCTGAGTCGTC
MPO	NM_000250	Forward Reverse	CAGCCCAGATATACCCCTCA GACAACACAGGCATCACCAC
NE	NM_001972.4	Forward Reverse	CATCGTGATTCTCCAGCTCA CTCACGAGAGTGCAGACGTT
COX-2	NM_000963	Forward Reverse	TCCCATGGGTTGTGTGTTTA AGATCATCTCTGCCTGAGTATCTT
IFNγ	NM_000619.2	Forward Reverse	TCCCATGGGTTGTGTGTTTA AAGCACCAGGCATGAAATCT
IL-1β	NM_000576.2	Forward Reverse	CTGTCCTGCGTGTTGAAAGA TTCTGCTTGAGAGGTGCTGA
TNF-α	NM_000594.3	Forward Reverse	TCCTTCAGACACCCTCAACC AGGCCCCAGTTTGAATTCTT
IL-6	NM_000600.4	Forward Reverse	TACCCCCAGGAGAAGATTCC TTTTCTGCCAGTGCCTCTTT
IL-8	NM_000584	Forward Reverse	TAGCAAAATTGAGGCCAAGG AAACCAAGGCACAGTGGAAC
MMP-1	NM_ 001145938	Forward Reverse	CTGCTTGACCCTCAGAGACC ATGCTGAAACCCTGAAGGTG
MMP-3	NM_002422	Forward Reverse	GAGTGTCGGAGTCCAGCTTC GCAGTTTGCTCAGCCTATCC
MMP-9	NM_004994	Forward Reverse	CAGGGATCTCCCCTCCTTAG GTCTTGTGGAGGCTTTGAGC
PPARγ	NM_005037	Forward Reverse	GCTGTGCAGGAGATCACAGA GGGCTCCATAAAGTCACCAA
GAPDH	NM_001289745.2	Forward Reverse	ACAGTCAGCCGCATCTTCTT ACGACCAAATCCGTTGACTC
HPRT	NM_002046.6	Forward Reverse	GAGTCAACGGATTTGGTCGT GACAAGCTTCCCGTTCTCAG

**Table 2:** Main composition of PF from VOO using COI/T20/29doc.

Phenol Composition	µM Phenol (50 µg PF/mL)
Hydroxytyrosol	41.07
Tyrosol	43.09
Vanillic acid	5.09
p-Coumaric acid	3.42
Decarboxymethyl oleuropein aglycone (dialdehyde) = oleacein	10.27
Tyrosol acetate	4.97
Decarboxymethyl ligstroside aglycone (dialdehyde) = oleocanthal	11.33
Pinoresinol	6.21
Cinnamic acid	6.89
Acetoxy-pinoresinol	6.22
Oleuropein aglycone, oxidized aldehyde form	39.18
Ligstroside aglycone, dialdehyde form	26.20
Luteolin	4.18
Apigenin	0.88

## Data Availability

The data presented in this study are available on request from the corresponding author.
